# Inclusive planning: African policy inventory and South African mobility case study on the exclusion of persons with disabilities

**DOI:** 10.1186/s12961-021-00775-1

**Published:** 2021-09-09

**Authors:** Marianne J. W. A. Vanderschuren, Obiora A. Nnene

**Affiliations:** Centre for Transport Studies, Department of Civil Engineering, University of Cape Town, Private Bag X3, Rondebosch, Cape Town, 7700 South Africa

**Keywords:** Africa, Disability, Mobility challenges, Transport, Policy, Planning

## Abstract

**Background:**

The Sustainable Development Goals (SDGs) and universal design (UD) principles call for inclusive planning. Within the transportation field, this includes the development or improvement of facilities that accommodate people with disabilities. Between 10% and 20% of the African population is affected by disabilities. A lack of understanding of the needs of people with disabilities leads to isolation. Within the transportation field, isolation manifests itself as a reduction in trip-making.

**Methods:**

This paper investigates the availability of transport policies and guidelines in 29 different African countries, focusing on the inclusion of persons with disabilities. A desktop study was conducted creating heat maps for 29 African countries, followed by the analysis of secondary data in the case study area, South Africa, demonstrating that the lack of adequate policies, guidelines, and appropriate implementation leads to a lack of accessibility, opportunities, and social isolation, measured through trip frequencies.

**Results:**

The data analysed revealed that many African countries omit, or only superficially include, people with disabilities in their transport policy framework. Ghana has the most inclusive People with Disabilities Act, while South Africa is most inclusive regarding their planning and design of transport facilities and services. In South Africa, 4.5% of the population did not travel at all in the 7 days before the interview, as disability or age prevented them from doing so, or due to a lack of appropriate travel services. When comparing the trip rates per week, people with disabilities travel significantly less, between 27.2% and 65.8%, than their abled counterparts.

**Conclusions:**

The study reveals that people with disability live less integrated, more isolated lives due to the lack of acknowledgement in the transport policy framework and accommodation in infrastructure and services. The results underpin the need for disability-inclusive planning in the African context and provide recommendations for actions that mitigate the isolation challenges faced by people with disabilities. Municipalities play a crucial role in improving the quality of life for people with disabilities.

**Supplementary Information:**

The online version contains supplementary material available at 10.1186/s12961-021-00775-1.

## Background

This paper investigates the inclusivity, or lack thereof, of transport planning for vulnerable population groups across a range of African countries, based on the analysis of available policy documents in selected African countries. Using South Africa as a case study, the paper then demonstrates the isolating effect that a lack of inclusive transport planning has on these vulnerable population groups and, in particular, persons with disabilities (PWDs), based on household survey data.

### Transport research on people with disabilities

Research challenges of transport planners demonstrate a shift in strategic priorities over time. In the 1970s, the focus was enhancing road capacity where drivers were predominantly male, middle-class workers, using private motor vehicles [29]. Four decades later, the focus has shifted to recognizing the needs of vulnerable transport user groups, highlighting the need to focus research attention on identifying gender issues in transport planning [2]. According to the authors, a further key area of research for the future is transport planning for PWDs.

Allen and Vanderschuren [2] identified the Transport Research International Documentation (TRID) database as the most inclusive data source. This is because the TRID database is an integrated source that combines the records from the Transportation Research Board’s Transportation Research Information Services (TRIS) database and the Organisation for Economic Co-operation and Development’s (OECD’s) Joint Transport Research Centre’s International Transport Research Documentation (ITRD) database. Hence, the database provides access to more than 1.25 million records of transportation research worldwide, including academic papers.

An analysis of publications in the TRID database over the past two decades across all modes of transport revealed a limited number of research publications focusing on PWDs. Based on the types of disabilities that affect the ability to move independently (i.e. disability, in general, hearing, vision and intellect/concentration impairment, as well as the use of mobility aids and epilepsy), a keyword search was conducted. Table 1 provides a summary of the number of publications found.

**Table 1:** Number of research publications relating to disability in the TRID database (2000–2020)

Keywords	Disability (general)	Mobility	Intellectual	Hearing	Vision	Epilepsy
Number of publications	83	12	12	12	1	1

Source: https://trid.trb.org/

It may be seen from the table that over the two decades analysed, research reports on transport-related challenges for PWDs were limited.

The United Nations’ (UN’s) general motto is to create peace, dignity, and equality on a healthy planet. The SDGs, established in 2015, unpack this motto further. SDG11, sustainable cities and communities, has 10 targets and 15 indicators. Target 11.2 states, “By 2030, access is provided to safe, affordable, accessible and sustainable transport systems for all, improving road safety, notably by expanding public transport, with special attention to the needs of those in vulnerable situations, women, children, PWDs and older persons” [45]. Despite the aspirations of the UN and SDG11, it can be concluded from the analysis of the number of transport-related research publications that studies on PWDs are underrepresented in mainstream research documents.

### Review of key publications

#### Disabilities in policies and legislation

Meriläinen and Helaakoski [26] found that inclusive transport is not (fully) considered in transport planning, design, construction, and implementation, especially in developing countries. This is in contrast with earlier findings by Metts [27], who concluded that “low- and middle-income countries now also have disability policies that reflect reasonably advanced concepts of disability, based on the UN 1982 World Program of Action Concerning Disabled Persons (WPA) and 1994 Standard Rules on the Equalization of Opportunities for Persons with Disabilities (Standard Rules)”.

The UN Convention on the Rights of Persons with Disabilities (CRPD), held in 2006 [43], recognized the importance for PWDs of their individual autonomy and independence, including the freedom to make their own choices, as well as the need for PWDs to have the opportunity to be actively involved in decision-making processes about policies and programmes.

Bardinard et al. [4] report that “accessibility is not yet a systematic concern in the planning or implementation of urban transport infrastructure” in East Asia and the Pacific, even though universal access principles originated in Japan. One of the implementation obstacles is the misconception that the application of universal design (UD) standards would be more costly [4, 34].

### Disabilities affecting independent mobility

“The transport justice [25, 37, 38] framework goes some way to link space and mobility in discussions about accessibility. However, it tends to overlook how people are differently embodied and how the interactions between the physical environment, including transport infrastructure, affects these people” [46]. UD, on the other hand, is the design and composition of an environment so that it can be accessed, understood, and used to the greatest extent possible, by all people, regardless of their age, size, ability, or disability (http://universaldesign.ie/What-is-Universal-Design/). According to the principles of UD, obstructions such as stairs, heavy doors, steep ramps, and poor signage/lighting should be minimized in the transportation system, to develop an environment that is truly open and functional to everyone [7]. However, barriers remain in governance, regulatory, planning, and implementation of universally accessible transport infrastructure and services.

Social exclusion for people with disabilities still exists. Part of this exclusion is due to a lack of funds for travel, as established by Khayatzadeh-Mahani et al. [20]. Other difficulties, due to long travel distances [32, 33], are exacerbated by a lack of or insufficient transport facilities and services. Mobility and access requirements of PWDs should be considered by planning and designing barrier-free transport systems. This implies an understanding and identification of the circumstances that create barriers for people with disabilities [26]. Equal access is often not provided in (public) transport planning as persons using mobility aids (crutches, a walking stick or a wheelchair) are confronted with many physical barriers, such as stairs in subway stations or inaccessible buses, when using the transport system [6, 14, 23]. Street and sidewalk conditions have a significant impact on persons with more severe impairment. The lack of and poor quality of footpaths, such as uneven surfaces due to cracks, were identified as a common barrier for people with vision impairment (VI) due to an increased risk of falling [12, 18, 35]. Facility maintenance or the provision of amenities can improve mobility independence almost immediately for someone who was previously unable to navigate transport facilities independently because of mobility impairment (MI) [10]. Venter et al. [47] reported, based on European, Asian, and African information, that a lack of UD implementation in urban transport leads to social difficulties, psychological pressure, and structural exclusion of people with disabilities. Curb cuts (depressed curbs that act as ramps in sidewalks), smooth pavement, and barrier-free sidewalks [21] are some of the environmental characteristics that can easily prevent mobility disability and promote independence in adults at greatest risk, such as those with underlying weakness in movement-related functions and balance. Yet, relatively little work has examined the effect of the built environment on mobility disability, particularly across those with different levels of physical impairment [9].

People with intellectual disability (ID), including aging people with cognitive impairment, commonly suffer severe communication limitations. However, written information continues to be the most common form of communication, creating notable access barriers [46]. These communications, and other barriers, require people with ID to rely on pre-booked support staff services, limiting their mobility and spontaneity in their social lives [28].

There are fewer transport barriers for persons with hearing impairment (HI), according to Chang et al. [8]. However, various studies have found that HI is associated with driving safety—increased crashes and poor on-road driving performance [11, 16].

Estimates suggest that disabled people in England and Wales undertake one third fewer journeys than “nondisabled” members of the population [1, 48]. Similar results were recently found by the authors during focus group interviews in Tshwane, South Africa.

### PWDs in Africa

Over one billion people globally live with some form of disability—about 15% of the world’s population, and this number is increasing. The number of people living with disabilities is expected to double to two billion by 2050 [49]. In countries with life expectancies over 70 years, individuals spend, on average, about 8 years, or 11.5%, of their life span living with disabilities (https://www.disabled-world.com/disability/statistics/).

Some 80% of PWDs live in developing countries, while an estimated 60–80 million of them are living in Africa. People with disabilities are estimated to account for 10% of the general African population, but the proportion may as high as 20% in the poorer regions. School enrolment for disabled minors is estimated at no more that 5–10% (https://www.disabled-world.com/news/africa/).

There is an apparent underreporting of disability in low-income countries, which has been attributed, in part, to the stigma associated with disability and the reporting methodologies used [5, 15, 31, 36, 39]. The UN Workshop on Disability (in Kampala during 2001) found that in many African societies, there are sociocultural pressures to underreport disability. Respondents are reluctant to admit the presence of PWDs in the household, and interviewers tend not to ask about disability unless a person with a very severe kind of disability is seen during the interview.

This lower reported prevalence rate is evident in South Africa, where the National Census of 2011 estimated the prevalence of disability to be 7.5% of the population [41]. Additionally, the highest prevalence of disability in the country has been reported among those with lower income, particularly those who had no schooling (10%) compared to those who had postsecondary education (3%) [41]. Black Africans in South Africa, who generally reside in under-resourced communities, were still found to have the highest rate of disability (7.8%) in the 2011 census [41].

**Fig. 1:**
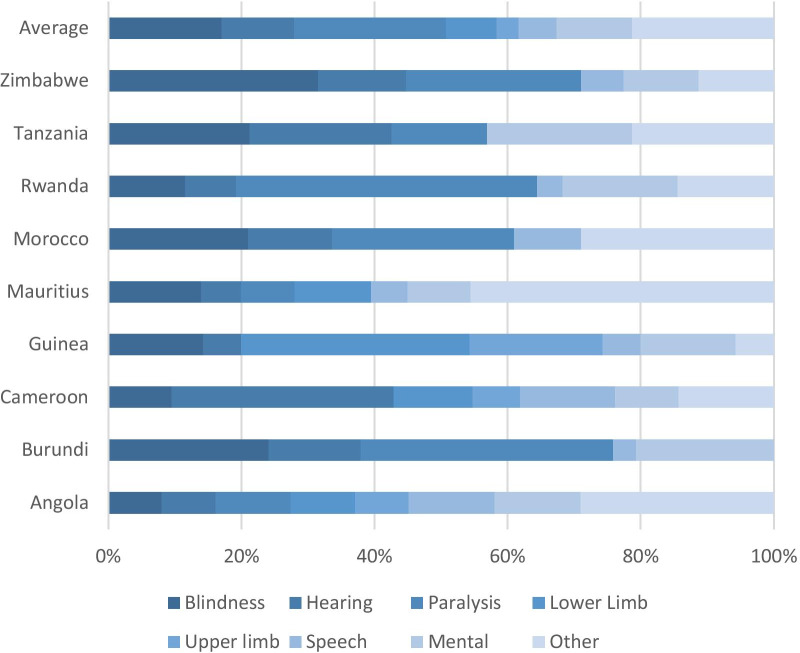
Types of disability in selected African countries. Source: UN Disability Statistics, accessed 30 August 2020

In the UN Disability Statistics database (https://unstats.un.org/unsd/demographic-social/sconcerns/disability/statistics/#/home), only 11 African countries report on disability levels. Of these, Senegal and South Africa do not provide a differentiation into types of disability. For all other available country data, the information is included in Fig. 1.

Most data included were from 2012 to 2014. The exception was Tanzania, with data from 2017. Underreporting, as identified by various sources, is also apparent in the UN disability statistics [44]. The countries that do report data, on average, report that 4.9% of their population live with disabilities. Exceptions are Tanzania and Zimbabwe, both reporting 9.1% of the population living with disabilities. Although these percentages are significantly higher than data for other African countries, they are still far below the 15% indicated by the Global Burden of Disease Report [50].

Furthermore, disability reporting categories are not standardized amongst African countries. Upper (4 countries) and lower (3 countries) limb-based disabilities are only reported by a limited number of countries. Cameroon and Guinea report zero paralysis cases, while Tanzania does not report any cases of speech impairment. These statistics, realistically, are highly unlikely.

Zimbabwe reports a significantly higher number of cases of VI (4.2%) and paralysis (3.5%), as well as the highest percentage of people with a HI (1.75%). Rwanda reports the highest level of paralysis cases (2.35%), while Tanzania reports the highest number of people with mental/learning disabilities (2.05%).^1^

Based on the literature, it can be concluded that PWD-oriented transport planning is highly encouraged on the African continent, given the vast number of affected individuals. Sources disagree about the actual level of inclusive planning in the developing world in general, and Africa more specifically. This paper enhances the knowledge on disability-inclusive transport planning in Africa through an inventory of the current planning document status quo. An analysis of mobility patterns in the South African context for PWDs provides insights into the level of isolation experienced by the most vulnerable in the South African society.

## Methods

The information in this study consists of two distinct parts, a desktop study of available transport policy and planning documents in African countries, and an analysis of secondary household data, for South Africa as a case study example.

The data for each country were collated into two distinct segments. The first segment relates to policy frameworks in the form of legislative and institutional support for PWDs within the countries. Here, documents such as the country’s constitution and other policy documents that address the living conditions of PWDs, with the aim of improving overall access to the various sectors of the economy, are collated and reviewed. The second segment of collated data indicates the availability of transport sector-specific provisions for particular types of disabilities within each country. A checklist comprising VI, HI, mobility aids, and other types of impairment was used to guide the data collection (Additional file 1: Policy Documents Raw Data).

The desktop study was conducted during the months of June and July 2020 by three researchers who were recruited for the purpose. Each researcher received training on the data type and collection method to be used before they commenced. The researchers rated documents on their inclusivity of PWDs compared to international best practices ([44]; https://nacto.org/). If more than one document dealt with a specific disability, the ratings were accumulated and assigned as scores to each country. The scores were then normalized to a scale of 0 to 10, using a linear normalization function (see Eq. 1).

1 $$x_{{{\text{normalized}} }} = \frac{{x - x_{{{\text{minimum}} }} }}{{x_{{{\text{maximum}} }} - x_{{{\text{minimum}}}} }}$$

This was done to ensure the uniformity of all the country data in terms of comparison and data visualization.

In total, 29 sub-Saharan African countries were surveyed: 11 Francophone and 18 Anglophone. The countries were from East, West, Central, Southern, and Northern Africa. The countries surveyed include Algeria (11), Benin (11), Botswana (4), Burkina Faso (10), Burundi (5), *Cameroon (8), Democratic Republic of the Congo (DRC) (11),* Cote d’Ivoire (10), Madagascar (10), Eswatini (1), Eritrea (4), *Ethiopia (4),* Gabon (9), Gambia (7), *Ghana (11), Kenya (9), Lesotho (2),* Liberia (1), Malawi (10), *Namibia (4), Nigeria (7),* Senegal (12), Sierra Leone (3), *South Africa (9), Tanzania (3),* Togo (4), *Uganda (5),* Zambia (11), and *Zimbabwe (3)*. Overall, 199 documents were reviewed. The number of documents per country are indicated in brackets. The practical knowledge and experience of the researchers in the local transportation context in 12 countries (indicated in italics) out of the 29 countries that were studied, was used as a basis to validate the data.

It should be noted that no contact was made with stakeholders in the various countries; as such, the data collection was limited to online available sources. This is identified as the main limitation of this study. Another limitation is that the accumulated count of policy documents, found on the disability areas highlighted in the checklist, was used as a measure of the extent of inclusiveness of each country. The authors acknowledge that the availability of documents alone is a limited metric to determine the disability inclusiveness of a country’s transportation policy. However, it was assumed that documents that were not available online^2^ would also be difficult to access and apply by local practitioners.

The second part of the study uses existing, secondary data for South Africa to assess the level of isolation for the vulnerable population groups identified, that is, PWDs. The South African National Household Travel Survey (SANHTS) raw data were used to conduct the analyses [42]. This database is the most comprehensive transport data currently available^3^ in South Africa. Data collection in this regard took place between January and March 2013, and a total of 51 341 households and/or dwelling units were sampled, using a random stratified sample design. Within the households, 157 273 respondents shared their transport information and opinions. Statistics South Africa, using a multitude of data available to them, created a weighting value for every household and person in the database to represent the whole South African population. All analysis in this paper applied the weighting.

## Results

In this section, the result of analysing the African country documents regarding their inclusivity of PWDs, in general, or UD specifically, is presented and compared to international best practices. The results also allow for a consideration of the level of isolation experienced by PWDs, as they are excluded from planning documents and institutional guidelines. Specifically, within the transportation field, this lack of inclusion manifests itself as a reduction in trip-making.

The document analysis results are presented as heat maps using Microsoft Excel for the visualization. For a given variable presented in the heat maps, a higher intensity, which is depicted by darker colours, indicates a higher score, hence, a greater level of inclusiveness for the given country in terms of that variable. The policy framework analysis and case study results are provided for the four themes that make up the earlier highlighted checklist, that is, VI, HI, MI individuals, and people with other impairments.

### VI

People with VI face the risk of being injured by obstacles and falling due to uneven surfaces. Furthermore, depending on the severity of the VI, the use of vehicles (both bicycles and private cars) is prohibited. Navigating the outdoors is a definite challenge for people who are visually impaired. The use of increased contrast, highly visible colours, and improved street lighting, the use of sound and tactile pavers, and the application of barriers and railings can improve the outdoor experience of people with VI.

**Fig. 2:**
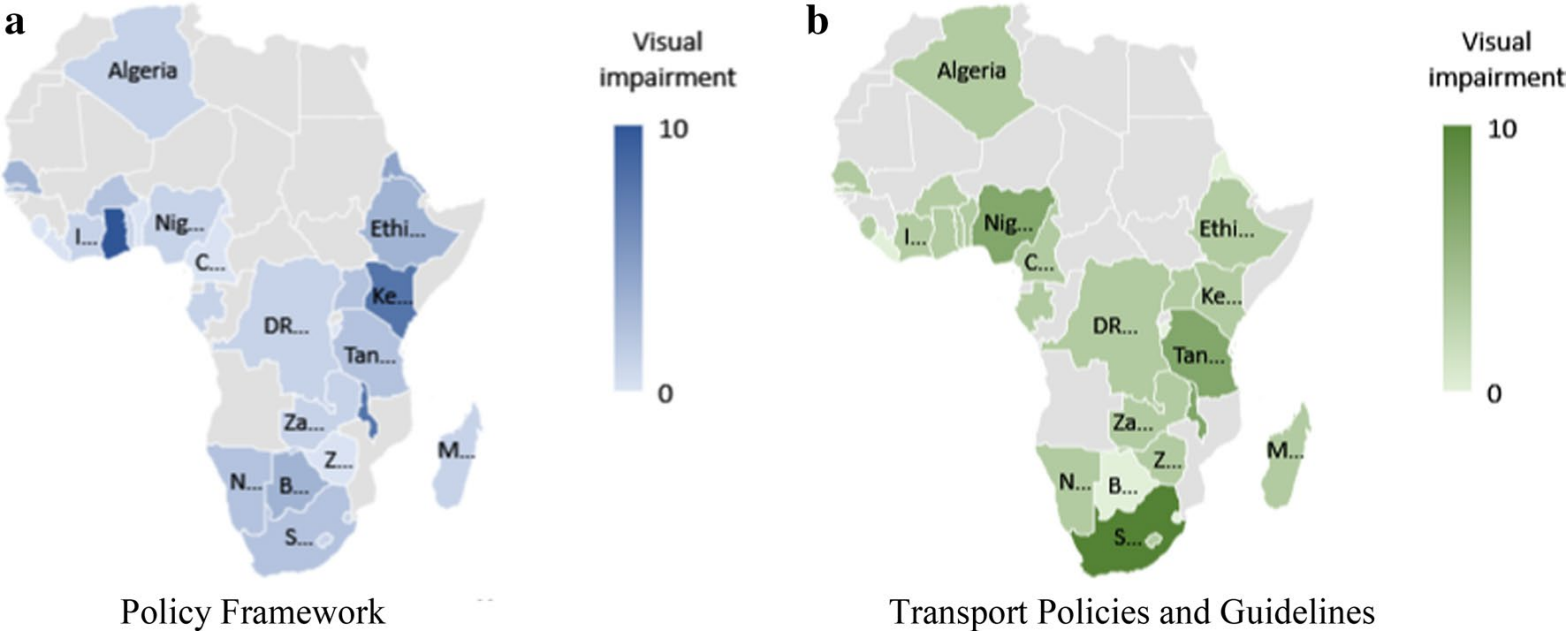
**a**–**b** Policy frameworks for people with VI. Powered by: GeoNames, TomTom, Wikipedia

In the African context, the recognition of VI is scarce. Of the 29 countries investigated, six do not mention VI in their policy documents (Benin, Burundi, Cameroon, Liberia, Sierra Leone, and Zimbabwe), while another 10 only mention the disability, but do not give any policy direction.^4^ The Ghanaian Persons with Disability Act 715 [13] is the most comprehensive document for people with VI, compelling social workers to start changing the physical space (see Fig. 2a).

The transport policy framework in eight investigated countries (Algeria, Botswana, Burkina Faso, Burundi, Eritrea, Liberia, Senegal, and Eswatini) do not include any accommodation of PWDs. The most inclusive transport-specific documents accommodating people with VI in the African context is found in South Africa. The documents legislate and guide the implementation of tactile paving, intersection design, and access to formal public transport (see Fig. 2b).

According to the SANHTS [42], South Africa has 4.15 million adults that have mild to severe VI, and this accounts for almost 15% of the population. In many cases, advanced prescription glasses can mitigate some of the negative impacts, improving the travel experience of the visually impaired. However, more severe cases of VI do experience reduced mobility, indicating a form of isolation.

In South Africa [42], over 2.2 million people (4.5% of the population) did not travel at all in the 7 days before the interview, as their disability or age prohibits them to do so, or due to a lack of appropriate services. For those that make trips, on average, the number of trips per person per week for people with VI is reduced by almost 40% [42].

**Fig. 3:**
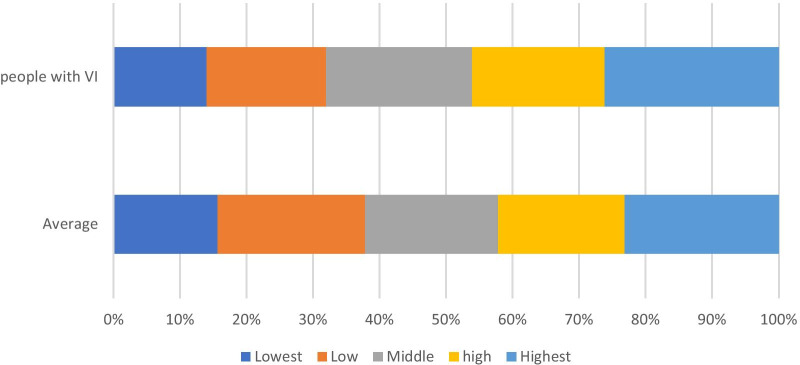
Trips per income quintile for able-bodied individuals and people with VI Source: SANHTS 2013

An analysis of the portion of trips for persons with/without VI per income quintile was conducted to understand whether the main reason for reduced mobility, and the resulting isolation, was disability-related, or whether other issues, for example, low household income, were at the core of this isolation. Although the distribution per income quintile is not identical between the two population groups, there is no significant bias towards any income quintile (see Fig. 3). The isolation experienced is due to the VI experienced.

### HI

Although less limiting, persons with HI do experience limitations when using the road network and transport services. In the Netherlands, a sign indicating a HI has been available for cyclists for over half a century to improve road safety. Scandinavian countries have similar signs, and there is research underway to improve and standardize the signs.

People with HI cannot anticipate traffic coming from behind, causing a hazard. Even crossing the road can be challenging, which has also been confirmed in research with people without HI, when vehicles are electric [30].

**Fig. 4:**
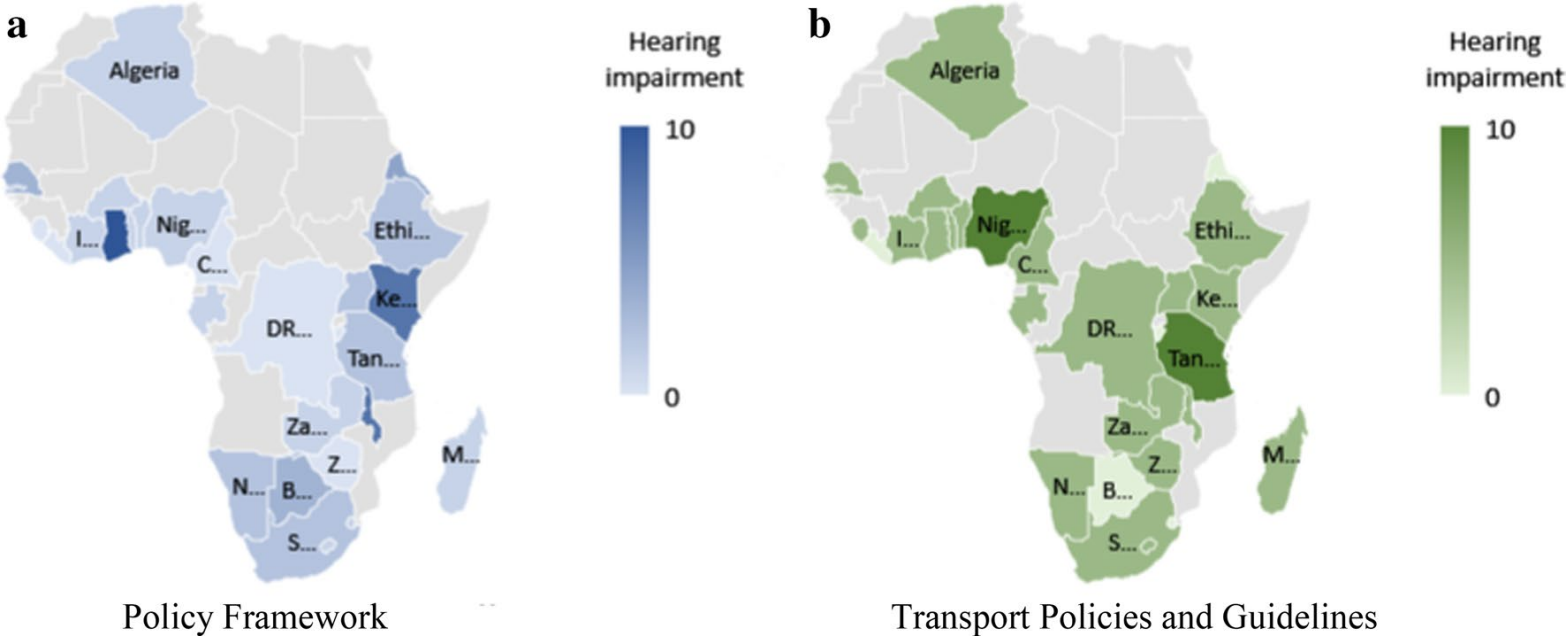
**a**–**b** Policy frameworks for people with HI

When analysing the policy frameworks in African countries, as displayed in Fig. 4a, again six countries (Burundi, Cameroon, DRC, Liberia, Sierra Leone, and Zimbabwe) do not mention HI in any of its policy and legislation documents. A further 10 countries only mention HI superficially. Ghana is again most inclusive to people with HI in its Act [13], followed by Kenya [19] and Malawi [24].

Figure 4b reflects the findings for the transport-specific policy framework. The previously mentioned eight countries do not include any HI attributes in their transport policy framework either. Nigeria and Tanzania are most inclusive regarding HI-related transport policies. However, research indicates that people with HI “appear to be the most vulnerable group in Nigeria and many other African countries” [17, 22]. Asonye et al. [3] found that children with HI are isolated from the public.

According to the SANHTS [42], 0.5 million people in South Africa have HI. The effect of this HI is that trip-making is reduced by 47%, which is, according to the authors, an indication of isolation. Analysing the distribution within income quintiles, as done previously for VI (see Fig. 3), did not provide a significant over- or underrepresentation for any of the population groups.

### MI

Experiencing MI has several causes. Most known are neuromuscular and orthopaedic impairments. However, people suffering from high blood pressure, obesity, asthma, and the like also experience compromised mobility abilities. People with MI can use various aids, such as a walking stick, crutches, or wheelchairs, to improve mobility.

**Fig. 5:**
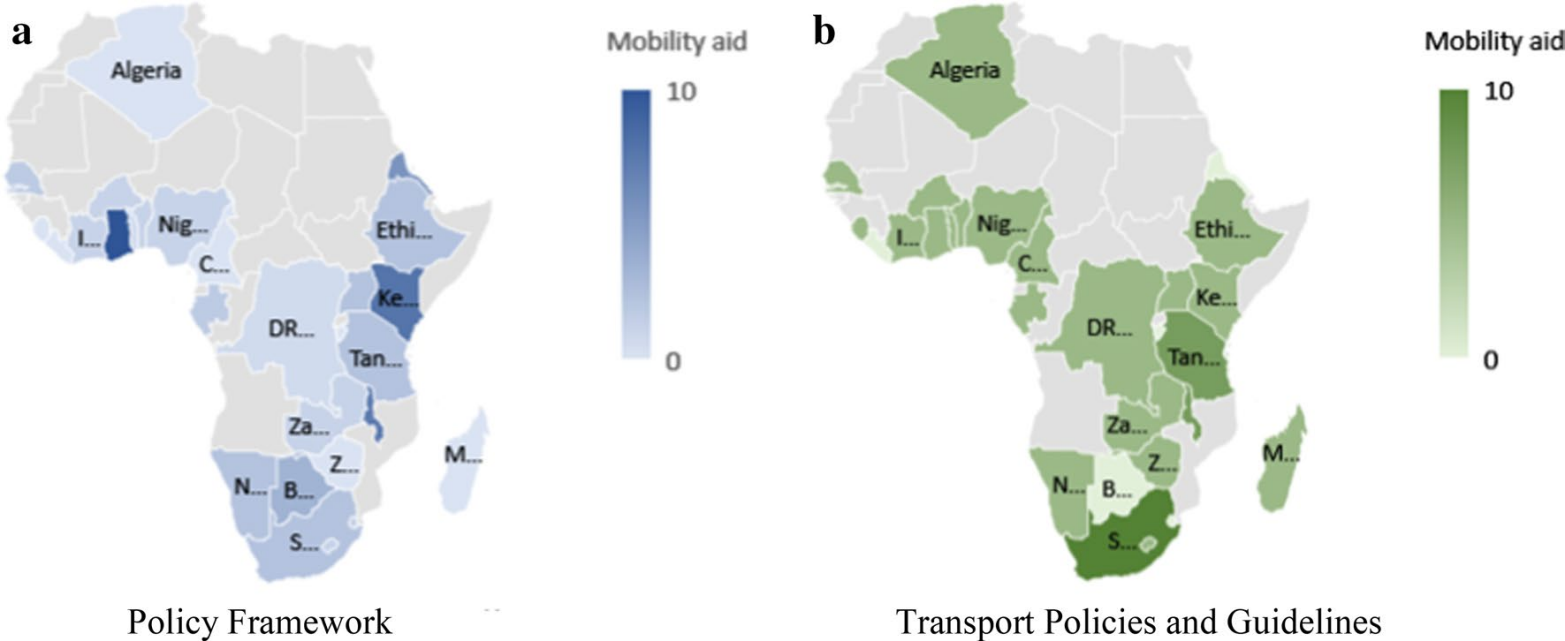
**a**–**b** Policy frameworks for people with MI. Powered by: GeoNames, TomTom, Wikipedia

In Africa, seven (Algeria, Burundi, Cameroon, Liberia, Madagascar, Sierra Leone, and Zimbabwe) of the 29 countries investigated do not mention any support for people with MI at all, including wheelchair users, in their policy frameworks. Another two countries only make a brief mention of persons with MI. Interestingly, though, the other countries have a reasonable inclusion of MI aspects in their policy frameworks (see Fig. 5a). MI is also included in the Ghanaian policy framework, followed by Kenya and Malawi (in that order).

Eight (Algeria, Botswana, Burkina Faso, Burundi, Eritrea, Liberia, Senegal, and Eswatini) of the 29 African countries make no allowance in their transport policy framework for people with a walking stick, on crutches, or in a wheelchair. All other countries, on the other hand, have a good to very good inclusion of MI in their transport policy framework (see Fig. 5b). South Africa’s transport policy documents are clearly superior regarding mobility aid requirements, followed by Tanzania and Malawi. South Africa specifies sidewalk surface requirements, the provision of drop curbs and intersection standards [40]. Furthermore, standards for the access of public transport are included.

**Fig. 6:**
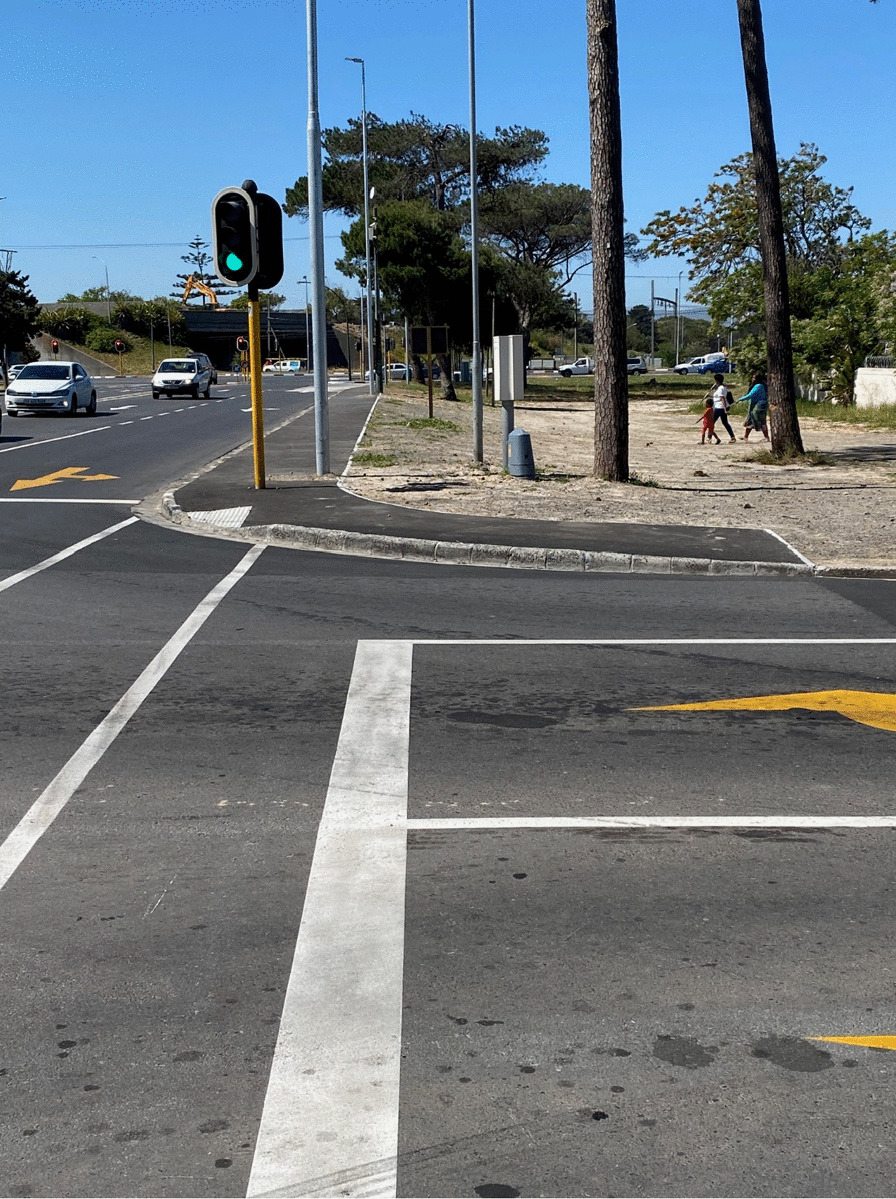
Picture of an intersection in Cape Town, refurbished in 2020. Source: Picture taken by author

Unfortunately, the inclusion of MI in the transport policy framework does not guarantee improved practice. A prime example is included in Fig. 6, where the drop curbs (with tactile paving) have been incorrectly implemented; the sidewalk in one direction is discontinued after approximately 10 m, and the traffic light is an obstacle for any wheelchair user.

The SANHTS [42] established that 0.70 million people in South Africa use a walking frame or stick to aid their mobility, while 0.12 million people are wheelchair users. The same database identified a significant reduction in trip-making of over 65% by people with MI. Recent focus group interaction with users of mobility aids (50% crutches and 50% wheelchair) confirmed the difficulties. Non-conducive surfaces and drop curbs, obstacles, high speeds, and aggressive traffic does make it impossible for this group to travel independently. Furthermore, over-dimensioned cambers add great difficulties  for people with MI even with a mobility assistant. In this qualitative data collection, the trip frequency of people with MI was 50% lower than any other vulnerable group. The MI focus group indicated that public transport is not conducive to travel, and that they will not make a trip if a private vehicle with a driver is not available. This at times even leads to students missing classes. Overall, people with MI indicate that they feel vulnerable when using the road environment, since they are slower than their able-bodied counterparts. This affects their road safety and personal security perception. Furthermore, they use more energy when moving, which can cause fatigue.

### Other impairments

Although the policy framework analysis did not yield specific information for people with concentration, self-care challenges, or memory impairment, when analysing the weekly trip rates of PWDs, compared to the average adult South African, trip-making reduced significantly. The analysis revealed that concentration impairment (−52.9%), self-care challenges (−27.2%), and communication impairment (−35.6%) reduce mobility and contribute to isolation. Again, household income does not influence these findings significantly.

## Discussion

The UN SDGs, more specifically Goal 11, and UD agencies call for more inclusive transport planning. Inclusive transport planning includes the accommodation of all road users, independent of gender, age, or ability. In this paper, the needs for PWDs have been unpacked. The literature provides a clear indication that transport is a burden for this population group. People with VI have the risk of falling and walking into obstacles. HI affects the anticipation of other traffic, which increases the road safety risk, while MI (people with a walking stick, on crutches, or in a wheelchair) requires more movement energy, and the slower movement also increases the road safety risk. Although some authors find that policy documents, specifically in developing countries, are reasonably reflective of advanced disability concepts, other sources disagree, concluding that there is a continued gap.

The findings from this study reveal that Africa still has a long way to go regarding the development and implementation of people-centric, inclusive transport planning. Many countries lack an appropriate conducive transport planning framework. Where general planning frameworks exist, such as in Ghana, the translation of the rights of PWDs is not translated into transport-specific policies and legislation. African countries must move towards a people-centric planning approach and translate this into the transport policy frameworks in respective countries. Furthermore, following the UN recommendation, PWDs should have the opportunity to be actively involved in the development of transport policies and programmes.

South Africa has the most inclusive transport policy framework that is inclusive of PWDs. However, this has not led to an extensive improvement in practice, although some good examples do exist. Based on an analysis of the SANHTS data [42], PWDs are likely to be at risk of isolation due to the lack of appropriate transport infrastructure and service provision. When comparing the trip rates per week, PWDs travel significantly less than their able-bodied counterparts. Their trip rates are between 27% and 66% less than their able-bodied, adult counterparts. Although various other socioeconomic factors also influence isolation, income was not significant for PWDs - in all income groups, PWDs make fewer trips.

Currently, across Africa, the lack of transport infrastructure and services to accommodate vulnerable road users, such as PWDs, which results from the lack of binding and enforceable policies, legislation, standards, and guidelines, serves to jeopardize vulnerable individuals’ safety, security, freedom, and, therefore, dignity.

Making sidewalks, public spaces, and public transport accessible to PWDs will also improve the transport system for other vulnerable groups, such as women, children, and the elderly. It is very likely that the improvement of transport infrastructure and services will catalyze the use of more environmentally friendly modes, including non-motorized transport and public transport.

## Conclusions

The literature related to PWDs is sparse, as established based on the inventory presented in Table 1, indicating a significant knowledge gap. When policy documents are reviewed, the inclusivity of PWDs is mostly conducted through content appraisal. This study applied the same technique, a decade after the last broad update on African countries.

Studies related to isolation analysis of PWDs are often qualitative. This study uses quantitative data to assess the transport isolation of PWDs. Notwithstanding the value of qualitative data collection, it is recommended that other countries and continents also establish whether common household surveys can provide improved insights on the lived reality of PWDs.

This study highlights the state of the transport sector in many African countries with respect to the integration of PWDs. A major issue identified in the study is the fact that due to the lack of consideration in the transport policy, institutional frameworks, and accommodation in infrastructure and services, people with disability live less integrated, more isolated lives. The results, therefore, accentuate the need for disability-inclusive planning and practice in the African context. Along these lines, recommendations are made for the improvement of African policy with the goal of mitigating the isolation challenges faced by people with disabilities. In the short term they are as follows:

- An improved understanding of the needs of PWDs can be gained from the analysis of existing databases, as demonstrated in this paper, as well as collecting new primary data. As PWDs are part of the most vulnerable in any society, resources reallocation towards needs assessment projects is key.
- Infrastructure audits will have to go together with improved financial practices, where contractors are paid a substantial part of the contract worth, after the UD aspects are signed off.
- The implementation of universally accessible infrastructure and services is complex and the “devil is in the details”. Community leaders, nongovernmental organizations (NGOs), lobby groups advocating for better transportation policies, and any other individuals or groupings representing the needs of PWDs should be included in ongoing transport infrastructure implementation projects. They can play a custodial role, assuring that investments are most effective on an ongoing basis.

Long-term, the following can be initiated:

- Countries need to make sure that their constitution, and other related policy documents, are people-centric, inclusive of PWDs and other vulnerable population groups. More inclusive countries have a PWDs act that describes the needs and rights of PWDs.
- Once the rights of PWDs and other vulnerable groups have been identified, a translation of these rights into the transport policy framework is required. People-centric policies and legislation, the adoption of UD standards, and the development of guidelines that approach UD practices in a holistic manner is a good start.
- African countries need to invest in the translation of inclusive transport policy frameworks into practice to address the isolation created for PWDs. This will require the strengthening of human resource capacity in municipalities where infrastructure investments are made. A further possibility is the creation of infrastructure audit capacity where new or refurbished infrastructure is assessed, based on UD practices before opening to the public.
- African countries need to address the road safety burden (as well as the personal security threats) experienced by vulnerable road users, including PWDs. Besides improved infrastructure, countries can apply other road safety measures, such as improved enforcement and education. Improving road safety and personal security will reduce the isolation experienced by PWDs.
- Community leaders, NGOs, and lobby groups play an important role in the African context, including in the transportation arena. However, this paper did not investigate their potential role. It is, therefore, recommended that further research is conducted in this field.
- Further studies are recommended that establish the impact on other fields, such as the environment, when improving transport infrastructure and services provided for PWDs and other vulnerable groups. Furthermore, the impact in other fields, such as access to education or jobs, should be estimated for PWDs and other vulnerable groups. Changing the way impacts are assessed will go a long way towards changing funding streams that are currently biased towards unsustainable, motorized, and private modes used by able-bodied individuals.

## Supplementary Information

**Additional file 1**. Policy Documents Raw Data.

## Data Availability

Data sources used during the desk top study are freely available online. The SANHTS can be sourced from the South African Bureau of Statistics.

## Abbreviations

CSSTL: Centre for Sub-Saharan Transport Leadership
HI: Hearing impairment
ID: Intellectual disability
ITRD: International transport research document
MI: Mobility impairment
MTL: Masters in Transport Leadership
OECD: Organisation for Economic Co-operation and Development
PWD: Person with disability
ReCAP: UKAid’s Research for Community Access Partnership
SANHTS: South African National Household Travel Survey
SDG: Sustainable Development Goals
TRID: Transport Research International Documentation
TRIS: Transportation Research Information Services
UD: Universal design
UN: United Nations
VI: Vision impairment

## Footnotes

1 The authors of the report warn that underreporting could have a significant impact on the reliability of the data in Fig. 1.

2 It should be noted that no contact was made with stakeholders in the various countries.

3 At the time this paper was written, the raw data for 2019 were not available to third parties.

4 Data on the policy document review, where PWDs were accommodated, are included in the uploaded database.
